# The Roles of Climate Change and Climate Variability in the 2017 Atlantic Hurricane Season

**DOI:** 10.1038/s41598-018-34343-5

**Published:** 2018-11-01

**Authors:** Young-Kwon Lim, Siegfried D. Schubert, Robin Kovach, Andrea M. Molod, Steven Pawson

**Affiliations:** 1 Global Modeling and Assimilation Office, NASA/GSFC, Greenbelt, Maryland USA; 2 Goddard Earth Sciences Technology and Research/I. M. Systems Group, College Park, MD, USA; 3 Science Systems and Applications, Inc, Lanham, MD USA

## Abstract

The 2017 Atlantic hurricane season was extremely active with six major hurricanes, the third most on record. The sea-surface temperatures (SSTs) over the eastern Main Development Region (EMDR), where many tropical cyclones (TCs) developed during active months of August/September, were ~0.96 °C above the 1901–2017 average (warmest on record): about ~0.42 °C from a long-term upward trend and the rest (~80%) attributed to the Atlantic Meridional Mode (AMM). The contribution to the SST from the North Atlantic Oscillation (NAO) over the EMDR was a weak warming, while that from El Niño–Southern Oscillation (ENSO) was negligible. Nevertheless, ENSO, the NAO, and the AMM all contributed to favorable wind shear conditions, while the AMM also produced enhanced atmospheric instability. Compared with the strong hurricane years of 2005/2010, the ocean heat content (OHC) during 2017 was larger across the tropics, with higher SST anomalies over the EMDR and Caribbean Sea. On the other hand, the dynamical/thermodynamical atmospheric conditions, while favorable for enhanced TC activity, were less prominent than in 2005/2010 across the tropics. The results suggest that unusually warm SST in the EMDR together with the long fetch of the resulting storms in the presence of record-breaking OHC may be key factors in driving the strong TC activity in 2017.

## Introduction

The 2017 Atlantic hurricane season was one of the most active on record. Based on statistics^1–3^, six major hurricanes developed, with two of them (Irma and Maria) reaching Category 5. The season is ranked as having the third highest number of major hurricanes in a single year over the past century, exceeded only by the 1961 and 2005 seasons. It is the first year since 1893 that 10 consecutive named storms have strengthened into hurricanes. A number of the tropical cyclones (TCs) that developed grew quite quickly to hurricane level and had unusually long life times (Harvey, Irma, Jose, and Maria). The accumulated cyclone energy (ACE) in the Atlantic, a measure of TC intensity and life cycle for the hurricane season, exceeded 220 × 10^4^ kn^2^, which is the fourth largest total ACE since 1950. The ACE for September 2017 (155.4 × 10^4^ kn^2^) is the largest value in a single month in the Atlantic basin.

The goal of this study is to identify the causes of the strong 2017 TC activity, with a focus on the long term trend and the leadingmodes of climate variability that impact seasonal TC activity over the Atlantic. Since almost all the unusual TC activity occurred during the months of August and September (AS), we focus our attention on those months. Previous studies have shown that climate variability influences TC activity through changes in both atmospheric circulation and thermodynamic conditions^4–7^. Specifically, the El Niño Southern Oscillation (ENSO) and the Atlantic meridional mode (AMM)^8^ are found to significantly modulate Atlantic sea level pressure (SLP) and deep convection throughout the tropics^9^. For example, La Niña and the positive phase of the AMM act to produce ocean/atmosphere conditions favorable for TC activity^10,11^. The AMM is known to exert interannual SST variations similar in structure to those of the Atlantic multi–decadal oscillation (AMO)^12,13^. In fact^13^, suggested that the impact of the AMO on seasonal TC activity manifests itself through the AMM. In addition to ENSO and the AMM, the North Atlantic Oscillation (NAO) also impacts TC activity^6,14,15^, with the negative phase favoring TC activity, and the positive phase suppressing TC activity: the latter was the case for the 2013 season, which was inactive despite above-average sea surface temperatures (SSTs)^16,17^.

SST variations associated with the climate modes described above (e.g.^18^) as well as the SST increase associated with long-term climate change, are well-known important factors that can enhance TC activity^19–22^. Ocean heat content (OHC) may, however, be a more important factor than SST for determining TC intensification^23^ as it measures the reservoir of heat available for maintaining high SSTs in the presence of mixing or Ekman pumping caused by TCs. Atmospheric impacts (both dynamical and thermodynamical) such as those associated with changes in vertical wind shear^24–26^, moisture^11^, atmospheric instability (e.g., convective potential energy)^27^, and tropical tropopause layer cooling^28,29^ over the Main Development Region (MDR) (80°–20°W and 10°–20°N) are also crucial factors for TC activity. The connection between tropopause layer cooling and TC intensity may, however, be weaker over the North Atlantic compared with the western Pacific^30^.

Here we focus on the factors that played an important role in producing the extremely active 2017 TC season, especially during August-September (AS). We examine the roles of three well-known modes of climate variability (ENSO, the AMM, and the NAO), along with the SST trend associated with a warming climate. We also compare the anomalous oceanic and atmospheric structures that occurred during 2017 with those observed during the other recent extreme hurricane years of 2005 and 2010.

## Results

### The 2017 North Atlantic SST anomalies

We begin by separating the SST anomalies over the North Atlantic during AS, the months when TC activity in 2017 was extremely active, into the contribution from the long-term linear trend and the contribution from interannual and longer-term variability.

**Figure 1:**
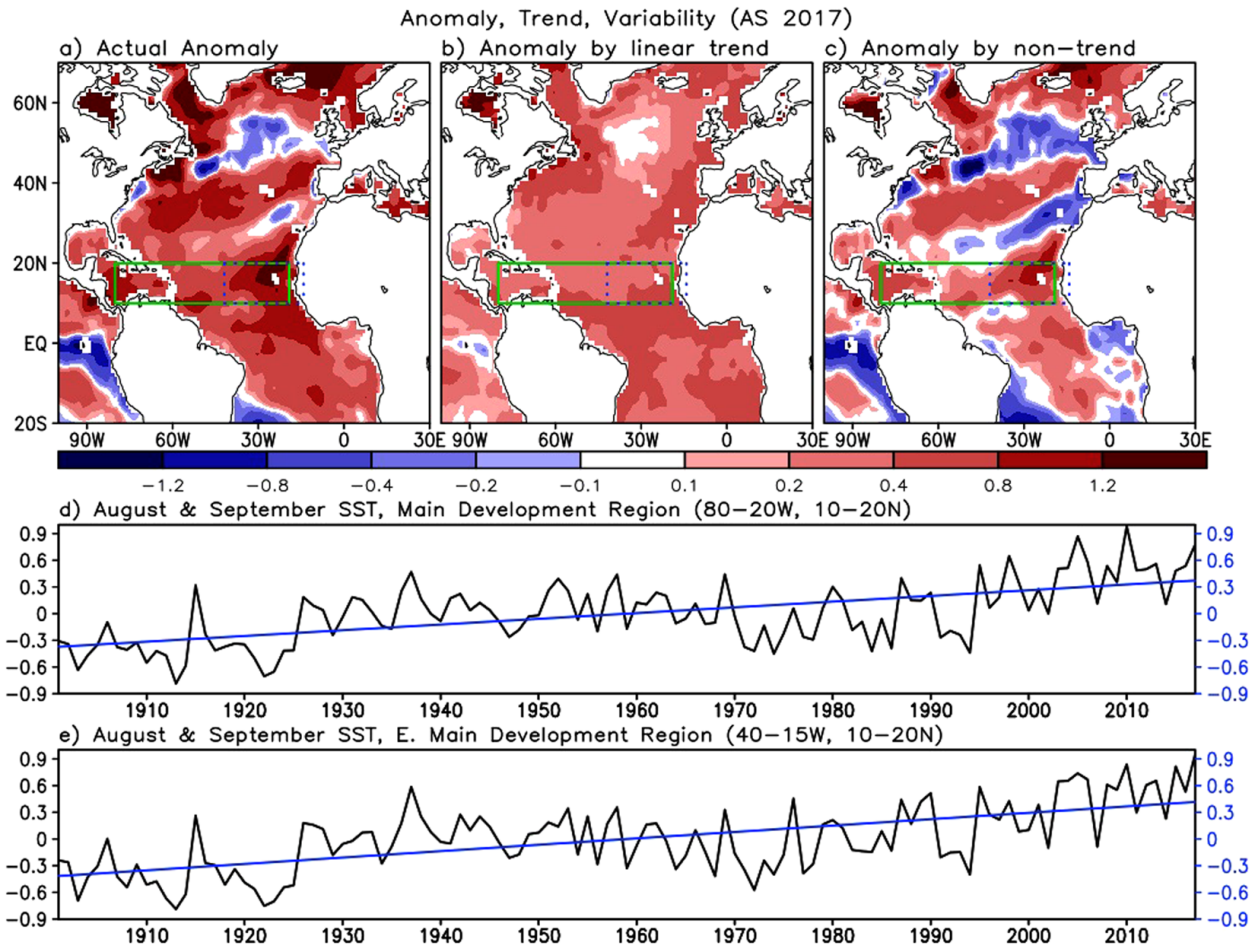
(**a**) Total SST anomaly from long term mean over 1901–2017, (**b**) anomaly by long-term linear trend, and (**c**) anomaly departing from the linear trend in August/September 2017. Green boxes denote the Main Development Region (MDR), while the blue boxes denote the eastern MDR (EMDR). (**d**) represents the area-averaged MDR SST in August/September over the period 1901–2017. Black and blue line denotes, respectively, the total SST anomaly (black) and long-term linear trend (blue). Bottom panel (**e**) is the same as the panel (**d**) but for the EMDR.

The three upper panels in Fig. 1 show the AS 2017 total SST anomaly, the contribution from the long-term trend (computed for the period 1901–2017), and the detrended anomaly that is presumably composed of interannual and longer-term variability. The distribution of the total anomaly (Fig. 1a) shows a large positive SST anomaly over the MDR with the maximum in the eastern Main Development Region (EMDR), along with both positive and negative anomalies over the mid-latitude Atlantic. The SST anomaly contributed by the long-term linear trend is positive throughout the Atlantic basin (Fig. 1b). The time series in the bottom two panels show the evolution of the SST anomalies over the MDR and the EMDR. The long-term trend contributions to the SST anomalies over the MDR and the EMDR region during 2017 are found to be ~0.37 and ~0.42 °C, respectively (blue lines in Fig. 1d,e). For comparison, the non-trend contributions are ~0.41 and ~0.56 °C over the MDR and the EMDR, respectively. It is evident from Fig. 1d that the MDR SST is the third highest in 2017, following 2010 and 2005. Interestingly, the SST anomaly in the EMDR region that includes Cape Verde, where several tropical cyclones developed and grew to be major hurricanes during AS 2017 (e.g., Harvey, Irma, and Jose), is the highest on record (~0.96 °C) (Fig. 1e). With a climatological value of 26.82 °C, this anomaly translates to a record warm SST value of ~27.8 °C in the EMDR region during AS, which should be quite favorable for TC development.

It has been suggested that, rather than the absolute SST in the Atlantic, a better indicator of hurricane activity might be the SST relative to that in the other ocean basins^31,32^. To assess whether 2017 is consistent with such an interpretation we present in Supplementary Fig. 1 the record of SSTs over MDR (upper) and EMDR (lower) relative to the global mean tropical SST. We find that although not exceptionally high, the relative SST anomalies are above average during the recent historic active years, such as 1995, 2001, 2005, 2010, and 2017. We note that the super active hurricane years that occurred in the 20th century (e.g., 1933 and 1969) also show higher than average SST, but very large relative SST anomalies are also found in 1945 and 1958, when the TC activity was not especially strong. As such, we might have to conclude that when considering the relative SST anomalies, there is less evidence that the 2017 SST would force an exceptional Atlantic hurricane season, though as^31^ point out, determining whether or not such an interpretation is correct will require further modeling studies and a fuller dynamical understanding of the tropical atmosphere. With that caveat, we will continue to focus here on the absolute SSTs.

### Impacts of the leading climate modes during AS of 2017

In order to assess the contributions of the leading climate modes to the SST anomalies during AS, we decompose the SST anomalies in terms of Rotated Empirical Orthogonal Functions (REOFs, see Methods). The three leading SST REOFs (captured as ENSO, the AMM, and an NAO-related SST pattern), are consistent with those found in previous studies (e.g.^6,9^) (see Supplementary Fig. 2 and related description) and account for ~65% of the AS interannual SST variance. We note that while the NAO is known to largely originate within the atmosphere, we interpret the REOF shown in Supplementary Fig. 2c as an NAO-forced SST pattern. The AMM (in its positive phase) stands out with warm SST anomalies across the MDR. Also, the AS SST anomaly over the MDR associated with ENSO is weak, consistent with^18^. We shall see, however, that the ENSO impact over the tropical Atlantic tends to be more strongly reflected in the vertical wind shear (Fig. 3).

**Figure 2:**
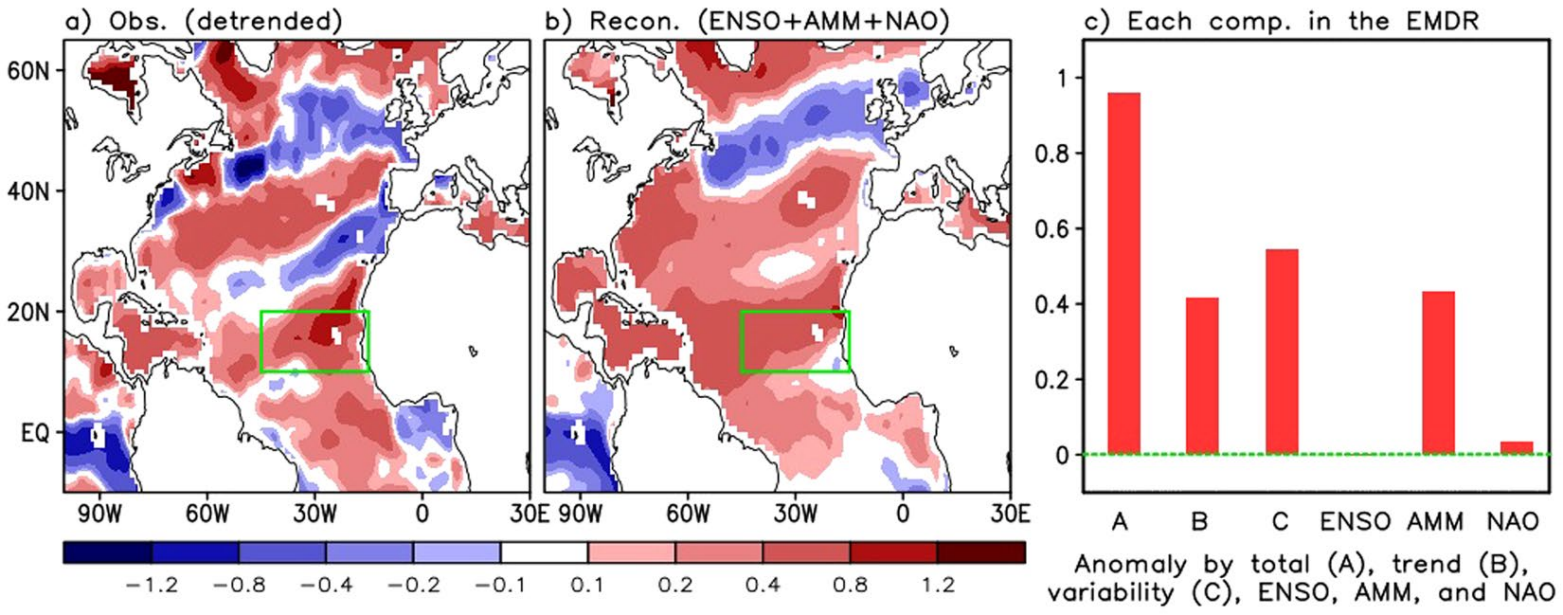
Distribution of (**a**) the observed SST anomaly (detrended component) in August/September 2017 and (**b**) the reconstructed SST anomaly by combined impacts of the ENSO, the AMM, and the NAO. Green boxes denote the eastern Main Development Region (EMDR). (**c**) Six bars in the graph in the right panel represent the SST anomaly over the EMDR by total, long-term linear trend, climate variation (i.e., departure from long-term linear trend), ENSO impact, AMM impact, and the NAO impact, respectively.

Having established the spatial structure of the leading modes of variability, we can reconstruct those aspects of the 2017 AS SST anomalies (i.e., departures from linear trend) (see Methods) associated with ENSO, the AMM, and the NAO. The left panel in Fig. 2 shows the 2017 AS detrended SST anomalies. The MDR is characterized by above-average SSTs, with the largest values in the EMDR. This tropical-wide distribution of the detrended warm anomalies, together with the contribution from the SST trend shown earlier (Fig. 1d,e), likely favors not only TC genesis but also the strengthening of the storms as they migrate westward across the Atlantic ocean toward North America. As mentioned earlier, the AS 2017 positive SST anomaly over the EMDR with respect to the 1901–2017 mean (0.96 °C), can be decomposed into a contribution from the linear trend (0.42 °C) and a contribution from interannual and longer term variability (0.54 °C): see the first three bars in Fig. 2c.

We next quantify the extent to which the detrended SST anomalies (Fig. 2a) can be explained by the leading climate modes of variability. Figure 2b show the reconstructed SST anomaly patterns, obtained by combining the contributions from ENSO, the AMM, and the NAO REOFs (see Methods). The results show that the reconstruction of the SST anomalies based on just the 3 leading REOFs (Fig. 2b) reproduces reasonably well (though not fully) the actual SST anomaly distributions shown in Fig. 2a. The bar charts (Fig. 2c) compare the SST anomalies over the EMDR with the anomalies reconstructed from the individual modes, showing that ~80% of the SST anomaly (0.54 °C, third bar in Fig. 2c) reflects the positive phase of the AMM (~0.43 °C, fifth bar in Fig. 2c) during AS 2017, with only a very weak positive contribution from the NAO (sixth bar in Fig. 2c). The contribution from ENSO, which was in a weak La Niña (or nearly neutral)^33^ phase during AS 2017, is negligible (fourth bar in Fig. 2c).

**Figure 3:**
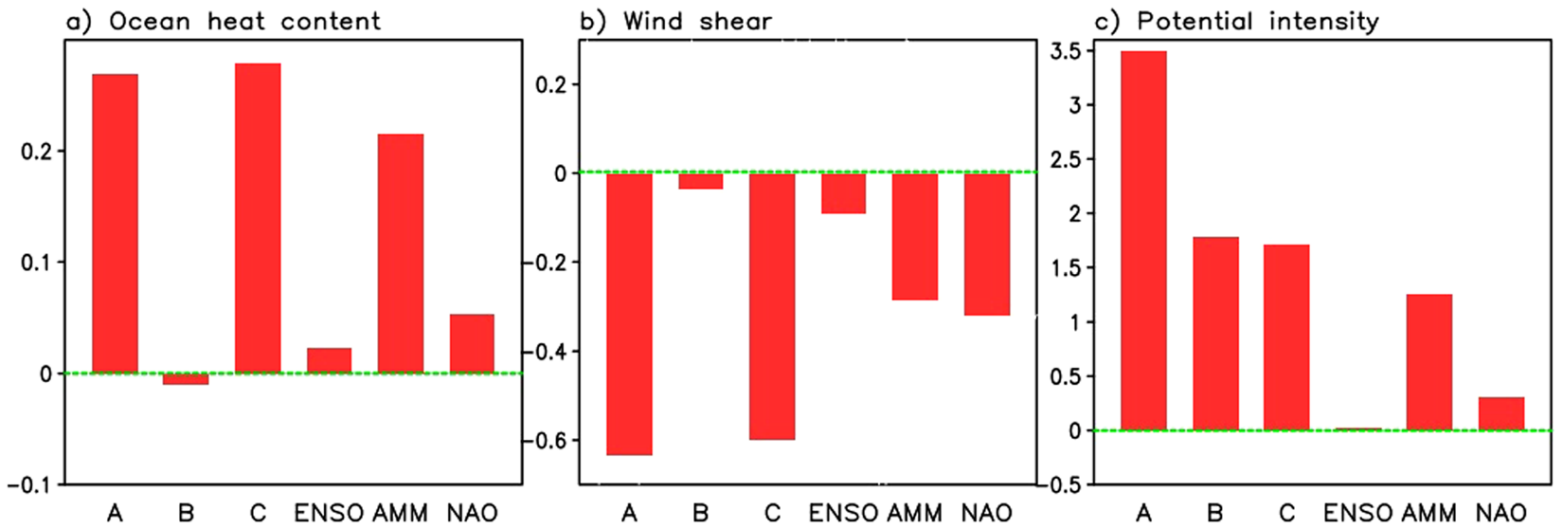
Anomalies of the (**a**) ocean heat content (10^22^ J) (ocean impact), (**b**) vertical wind shear (m s^−1^) (dynamical impact), and (**c**) potential intensity (m s^−1^) (thermodynamical impact) over the eastern Main Development Region by total (A), linear trend (B), detrended (C), ENSO impact, AMM impact, and the NAO impact in August/September 2017. Note that detrending was done for the data available period 1982–2017.

We next extend our investigation to examine ocean heat content (OHC), and the dynamical and thermodynamical aspects of the atmosphere known to impact TC activity. Figure 3 presents the anomaly by total (A), linear trend (B), detrended (C), and the reconstructed anomalies associated with the individual modes for OHC (left) (ocean impact), wind shear (middle) (dynamical impact) and potential intensity (PI) (right) (thermodynamical impact) in the EMDR for the period 1982–2017 (see Methods). Here the magnitude of the vertical wind shear is defined as

1 $$Wind\,shear=\sqrt{{({U}_{850}-{U}_{200})}^{2}+{({V}_{850}-{V}_{200})}^{2}},$$

so that both westerly and easterly vertical shear have positive values. Thus, smaller wind shear magnitude corresponds to larger negative anomalies as defined here.

The results show that that PI has a substantial upward trend over the EMDR region,while the OHC and wind shear do not. As such, the total anomaly of the PI consists of both a trend and detrended components (Fig. 3c), while the total anomalies of the OHC and wind shear differ little from the detrended anomaly over the EMDR region (Fig. 3a,b). Taking a broader look (Supplementary Fig. 6b), we find that there are regions west of the EMDR where the OHC does have a substantial upward trend component, especially over the western to central extra-tropical Atlantic.

The most striking feature associated with the leading modes is that they drive higher OHC, weaker wind shear and a vertically more unstable atmosphere than average in AS 2017. For example, the sum of reconstructed anomalies of OHC, wind shear, and PI over the EMDR from individual modes is about 0.29, −0.58, 1.5 (the right three bars in Fig. 3a–c), respectively, demonstrating their positive impact on TC activity in AS. We also see that the reconstructed anomalies are generally close to the detrended anomalies, which are 0.28 for OHC (the third left bar in Fig. 3a), −0.6 for wind shear (the third left bar in Fig. 3b) and 1.7 for PI (the third left bar in Fig. 3c). Comparing the impacts of each mode highlights that the AMM is the key factor driving the ocean and thermodynamic impacts (Figs 2 and 3a,c). In contrast, the wind shear, known to also be dynamically linked to the jet stream, atmospheric pressure and circulation fields associated with ENSO and the NAO, is influenced by all three climate modes (Fig. 3b).

### Comparisons with other years

In this section, we compare the spatial distributions of various key physical quantities such as vertical wind shear and SLP (dynamical impact), PI and outflow temperature (thermodynamical impact), and SST and OHC (ocean impact) in AS 2017 with those during previous extremely active hurricane years (e.g., 2005 and 2010). For each of these quantities, the ranking is calculated at each grid point for the years 1995–2017 - the recent period of above-average TC activity^34,35^. Note that SLP and vertical wind shear and outflow temperature values are ranked in the order of low to high, because lower SLP, weaker wind shear, and lower outflow temperature associated with tropical tropopause cooling facilitate strong TC activity, while the remaining quantities are ranked from high to low, because warmer and higher potential energy conditions (moister and more unstable) are favorable for TC activity. Additionally, standardized SST and OHC anomalies are compared among the three years in a quantitative manner in the middle part of this section.

**Figure 4:**
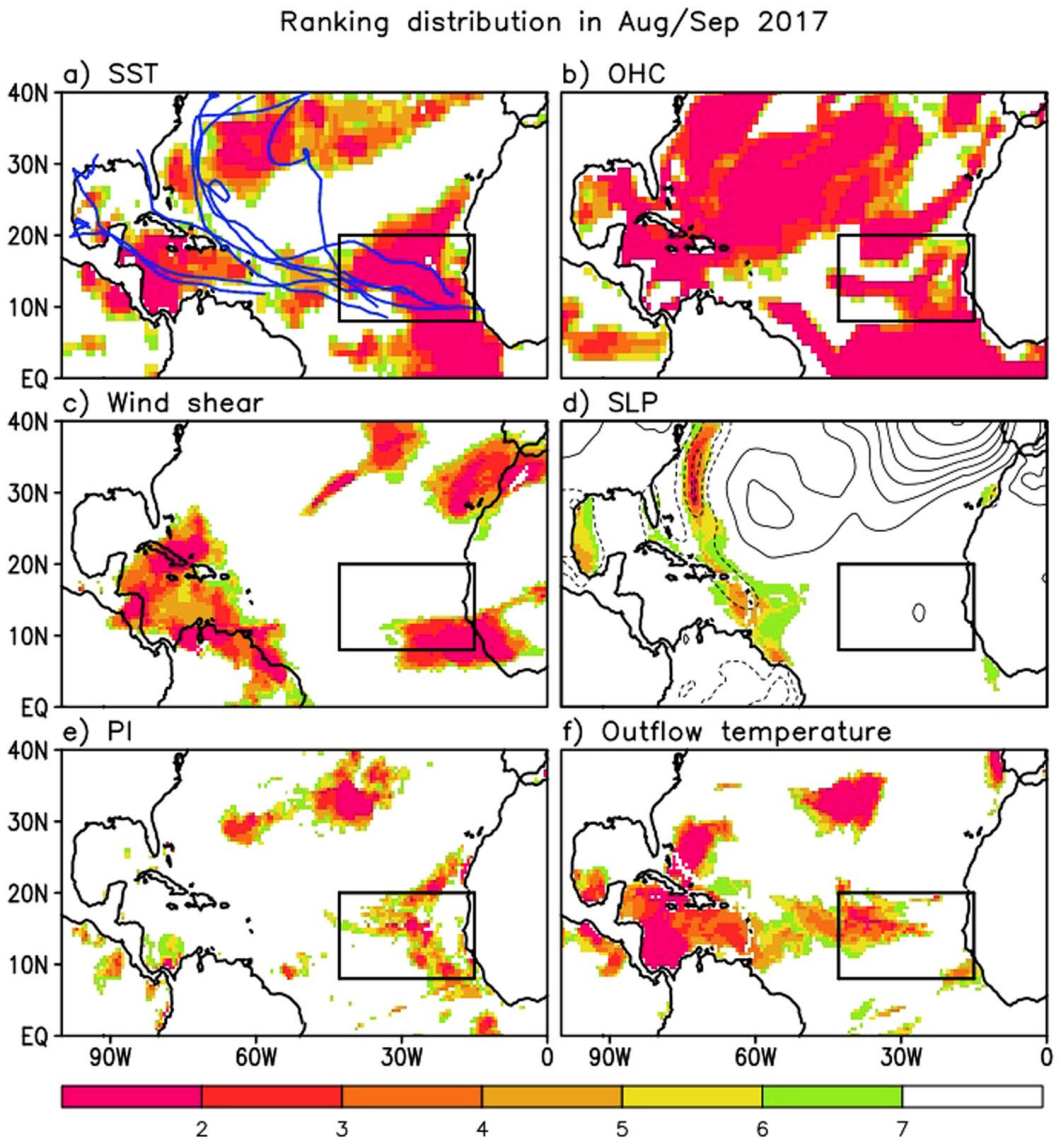
Distribution of rankings corresponding to the anomalies of each key quantity during August/September 2017. Rankings are calculated over 1995–2017, the recent period of above-average tropical cyclone (TC) activity on decadal time scale. Ranking values are shaded only for the first top six rankings (1st – 6th). Key quantities investigated here, which play a crucial role in TC activity, are SST and ocean heat content (ocean impact), vertical wind shear and sea level pressure (dynamical impact), potential intensity and outflow temperature (thermodynamical impact). The blue lines in (**a**) are the TC tracks observed in August/September 2017. Contour lines in (**d**) represent the sea level pressure anomaly distribution. Black boxes denote the eastern Main Development Region (EMDR).

Figure 4 depicts the distributions of the rankings for AS 2017. The EMDR (an area of substantial TC genesis during AS 2017) has extensive areas for which AS 2017 is ranked in the top three for SST, OHC, and wind shear (Fig. 4a–c), while this is not the case for SLP. High rankings over the EMDR are also found for PI and outflow temperature. Specifically, OHC, a vital factor that can boost the rapid intensification of hurricanes^23,36^, is ranked in the top (or close to top) over most of the Atlantic basin, indicating its important role in 2017. The rankings for wind shear and outflow temperature (Fig. 4c,f) tend to show high rankings over much of the Caribbean Sea and northward to just north and east of the Bahamas. The highest rankings for SLP occur in narrow swaths along the east coast Mexico, and from the southern Caribbean Sea northward along the east coast of North America. Figure 4 overall indicates that a number of key quantities provide very favorable conditions for TC activity in AS 2017, especially over the EMDR. Ocean conditions appear to be even more favorable than atmosphere conditions (as measured by the rankings) for developing intense TCs.

**Figure 5:**
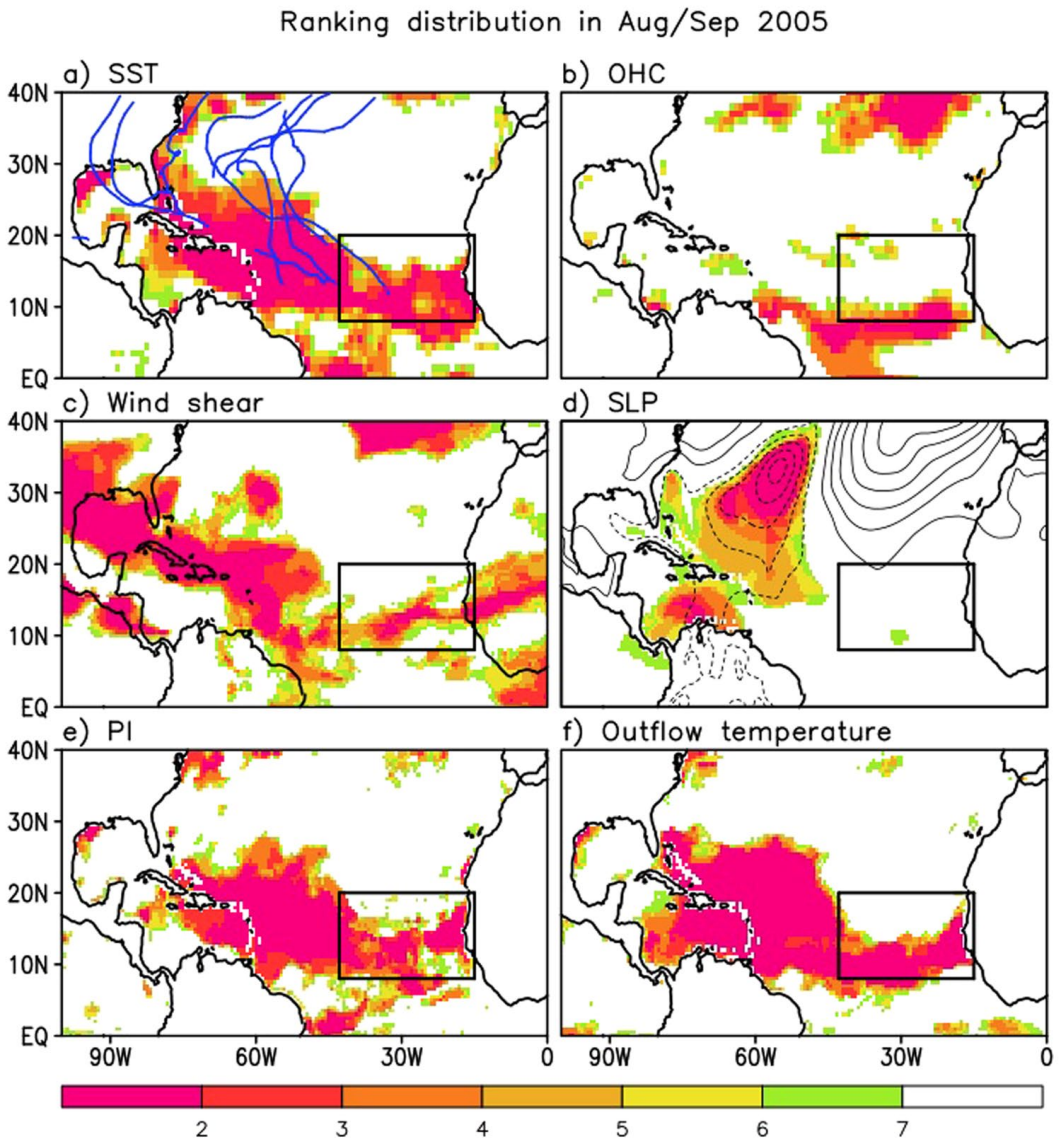
Same as Fig. 4 but for the other extremely strong hurricane year that occurred in 2005.

The above results for 2017 are next compared with those that occurred in 2005 and 2010. Figure 5 shows that during 2005, highly favorable thermodynamical conditions (Fig. 5e,f) were widespread across the tropical North Atlantic. This is in contrast to 2017 during which the most favorable conditions were mainly confined to the EMDR. On the other hand, the impact of the ocean during 2017 is comparable to or even stronger than that which occurred in 2005. Specifically, OHC, that can act as a reservoir to keep the ocean surface warm, is substantially higher in ranking during 2017 than in 2005 (cf. Figs 4b and 5b). A comparison with 2010 (Supplementary Fig. 3 (SF3)), reveals that 2017 also had more favorable OHC conditions than that year. However, during 2010, atmospheric conditions (SF3c–f) were more favorable for TC intensification throughout the tropical North Atlantic.

Supplementary Figs 4 (SF4) and 5 (SF5) compare the SST and OHC between the three years in a more quantitative manner (by providing amplitude information) in order to support our conclusions drawn from ranking analysis. By scaling the SST anomalies in terms of standard deviation, those figures show that each year has a different spatial distribution of where the SST anomalies are largest (greater than 1 or 2 standard deviations), and those regions tend to be juxtaposed with the regions of TC genesis and evolution (SF4): see also^17,37^. For example, the largest SST anomalies are confined to the west in 2005 and to the east in 2017, while 2010 shows large SST anomalies over much of the MDR. Similarly standardized OHC anomalies (SF5) match well the rankings shown in Figs 4b and 5b, and SF3b, supporting our contention that the three years have unique OHC distributions, causing different impacts on TC activity.

Earlier studies concluded that the main TC tracks on seasonal time scales are significantly determined by the combined impact of the ENSO, AMM, and NAO that characterize the SLP distribution^9,17^. Comparing the SLP anomaly patterns for the three extreme years 2005, 2010, and 2017 (see Figs 4d and 5d, and SF3d), the 2005 season, characterized by the largest positive AMM and near neutral ENSO and NAO conditions^17,38^, had the lowest SLP (and high ranking) largely confined to the western/central North Atlantic (consistent with the TCs that developed there and moved northward): the area of low SLP did extend south to just north of Cuba where, in the presence of favorable wind shear, a number of TCs developed that made landfall over North America (Fig. 5a). During 2010, the combined impact of the strong positive AMM, La Niña, and the negative NAO^38^ produced positive SST anomalies across the entire tropical Atlantic and a weaker subtropical high (i.e., higher SLP ranking) over the Atlantic (SF3d), leading to more early recurvers and thus fewer landfalls despite enhanced TC genesis^37^ (SF3a). This is in contrast with 2017 that has TC tracks directed westward toward the Caribbean Sea with landfalls over North America due to development of the subtropical high not unlike what occurred in 2005 in the Atlantic (Fig. 4a,d).

The above results indicate that it is to a large extent the differences in the phases and intensities of the three leading climate modes that determine the unique TC track patterns observed during these three (2005, 2010, and 2017) strong TC seasons. In particular, the overall very favorable ocean/atmospheric conditions for TC activity is linked to the large amplitude positive phase of the AMM in those years (see the PC and AMM index in Supplementary Fig. 2e).

The reasons for the differences between 2017 and the other two extreme years are not immediately clear. The relatively larger amplitude of the AMM during 2005 and 2010 compared with 2017 (Supplementary Fig. 2e) appears to be why the highly favorable atmospheric conditions for strong TC activity extended across much of the North Atlantic during those years while that was not the case for 2017. On the other hand, the more favorable OHC conditions in 2017 compared with 2005 and 2010 appears to be associated with the increasingly more important role of the trend, as computed here for the period 1995–2017 (Supplementary Fig. 6b). This upward trend is primarily observed over the western-central North Atlantic and, unlike for the atmospheric quantities (Supplementary Fig. 6c–f), has a distribution that is quite similar to the distribution of the 2017 OHC rankings (cf. Fig. 4b). As such, it appears that the larger OHC in 2017 is the combined effect of the trend (most pronounced over the western-central North Atlantic) and the leading modes of climate variability including the AMM that have influences spanning the North Atlantic.

Further evidence of the important role of the AMM over the North Atlantic is presented in Supplementary Figs 7 and 8 (SF7 and SF8). In particular, the spatial correlations between the observed anomalies of some key variables in 2017 and the corresponding anomalies determined from a regression against the AMM (SF7), provide evidence of a strong association between the ocean/atmospheric anomalies and the AMM. Looking at the longer record (1995–2017; see SF8 and related discussion), it is clear that the AMM is also closely related to the interannual variation of the number of major hurricanes (SF8e). These facts suggest that the anomalous spatial patterns of the rankings in Figs 4 and 5, and SF3 have a close relation to the AMM, though ENSO and the NAO play a role as well. We note that the higher correlation of the ocean/atmospheric anomalies (e.g., SST, SLP, wind shear, humidity, and atmospheric instability) during TC season with the phase/amplitude of the AMM than with either ENSO or the NAO, has been reported in previous studies^13^.

## Discussion

This study examined the causes of the extremely strong 2017 Atlantic TC activity, focusing particularly on AS when much of the activity occurred. A key factor suggested was the record-setting warm SST over the EMDR, driven primarily by the climate change signal (~0.42 °C above the 1901–2017 average) and the AMM that accounted for 80% of the additional (beyond the trend) warming of ~0.54 °C. As such, a majority of the tropical disturbances that developed into strong TCs (Gert, Harvey, Irma, Jose, Lee, and Maria) had their genesis in the EMDR. In addition, the MDR had the third warmest SST on record exceeded only by 2010 and 2005. This was accompanied by record-setting OHC over most of the North Atlantic that acted to maintain the warm ocean surface and facilitated the strengthening of the TCs as they traversed the Atlantic. Atmospheric conditions (e.g., wind shear, SLP, PI, and upper-level outflow temperature) also provided very favorable conditions for TC activity over the Atlantic with the maximum over the EMDR, but these factors were overall less prominent than in 2005 and 2010 across the entire Atlantic basin. ENSO, the NAO, and the AMM together provided the favorable wind shear conditions, while the AMM also produced the very warm ocean and enhanced atmospheric instability.

While we believe the results of our observational analysis are highly suggestive of the causes of the 2017 extremely strong TC activity as summarized above, a natural follow-up step is to carry out model experiments that would allow a more direct assessment of the nature of the remarkably warm SST and OHC due to both climate change and climate variability, as those experiments are found in^39–42^. Such experiments would likely require a model that is coupled to the ocean (rather than an AGCM) to allow addressing the role of OHC, and has high enough resolution to address the possible roles of spatial (and temporal) scales smaller (shorter) than those considered here, including the possible role of African easterly waves.

Understanding the implication of these results for the future requires that the 2017 hurricane season be considered in the context of past seasons. Simply assuming that the SST continues to increase for the next few decades due to global warming, some enhancement of seasonal TC activity can be expected, including the development of hurricane–level TCs^20–22,43^. Also, the downward trend of temperature near the tropical tropopause in recent decades and the associated cooling of the TC outflow temperature appears to contribute to an increase in TC PI^28,29,44^. On the other hand, seasonal TC activity over the past few decades displays considerable interannual variability that is largely determined by the leading modes of climate variability, indicating that a gradual warming alone does not play the dominant role^45,46^. In fact, the most extreme TC seasons in the recent past tended to occur when these modes of climate variability provide favorable conditions for TC activity (e.g., 2005 was characterized by a very strong positive AMM and 2010 was characterized by a positive AMM and La Niña conditions). In contrast, the recent weak TC activity in 2014 and 2015, for example, occurred in the presence of El Niño conditions during summer, which would tend to suppress TC activity. The weak TC activity with many short TC tracks somehow coincided with a positive phase of the NAO in 2013, while the ENSO and AMM signals were rather weak. Strong anti-cyclonic Rossby wave breaking, which tends to be more active during the positive phase of the NAO^47^, was also observed during AS 2013^48^, driving an equatorward intrusion of extratropical dry air. TC development was below normal during AS 2016, despite warm Atlantic conditions, due to an anomalously dry troposphere over the MDR^49^.

The above cases indicate that, even in the presence of climate change characterized by increasing SST, it is the leading modes of climate variability that largely determine the extremes in seasonal TC activity, in that they are associated with both the thermodynamical and dynamical conditions favorable (or unfavorable) for TC development. Nevertheless, we can expect that climate change will play an increasingly important role in determining extremely active years in that it provides an increasingly warmer baseline in SST from which the major modes of climate variability deviate. The 2005 and 2017 hurricane seasons (both characterized by a positive AMM, and weak NAO and ENSO) appear to be consistent with such an interpretation. During those years, the tropical Atlantic SSTs and the major hurricane counts are comparable, despite a relatively smaller magnitude of the positive phase of the AMM in 2017 than in 2005 (e.g., Supplementary Fig. 2e), indicating an increasingly greater role for climate change.

## Data and Methods

The SSTs used are the Merged Hadley-NOAA Optimal Interpolation SST data^50^ at 1° longitude-latitude resolution over the period 1901–2017. The atmospheric data (0.625° longitude × 0.5° latitude resolution) are from the NASA Modern-Era Retrospective analysis for Research and Applications, Version 2 (MERRA–2)^51^. The primary MERRA–2 variables used are SLP, 500 mb vertical velocity, and the three dimensional horizontal wind, relative humidity, geopotential height, and temperature, at 25 pressure levels (100–1000 mb)^52^. The study also uses the ocean heat content (300 m) (OHC) derived from Version 1 of the NASA Global Modeling and Assimilation Office Ocean Data Assimilation System (GMAO ODAS)^53^.

TC track data are employed to show and compare their characteristic patterns between 2005, 2010, and 2017. The data are downloaded from NASA EarthData Global Hydrology Resource Center (GHRC)^54^.

In order to capture the leading modes of climate variability that play a major role in determining interannual variation of the ocean/atmosphere, the Rotated Empirical Orthogonal Function (REOF) analysis technique^55^ is applied for the AS months over the period 1982–2017. Specifically, varimax rotation method is applied so that the REOF modes can meet orthogonality to each other. We extract the leading REOF spatial patterns (left panels in Supplementary Fig. 2 (SF2)) and corresponding time series (black lines in the right panels in SF2) from the detrended SST anomaly data. The time series (Principal Component time series) present interannual variation of each mode. The time series in blue denote official indices of the ENSO, the AMM, and the NAO archived at NOAA Climate Prediction Center (for ENSO^56^ and NAO^57^) and University of Wisconsin (for AMM^58^).

In order to examine how much of the detrended anomaly in 2017 is explained by a combination of ENSO, the AMM, and the NAO modes, the anomaly is projected onto the REOFs of each mode. For example, the reconstructed SST_ENSO_ (x, y, t) for the ENSO mode at (x, y) and time t is then

2 $${{\rm{SST}}}_{{\rm{ENSO}}}({\rm{x}},{\rm{y}},{\rm{t}})={{\rm{R}}}_{{\rm{ENSO}}}({\rm{x}},{\rm{y}})\cdot {{\rm{PC}}}_{{\rm{ENSO}}}({\rm{t}}),$$

where R_ENSO_ (x, y)is the unnormalized REOF SSTs for the ENSO mode and PC_ENSO_ (t) is the normalized (detrended) PC time series. This calculation is repeated for the other two modes, which are orthogonal to each other, over 1982–2017. This procedure helps quantify the effectiveness of the leading modes in reconstructing the observed anomaly each year.

To assess the atmospheric heat potential that determines atmospheric instability, we calculate the potential intensity (PI) (*V*_*pot*_) following^59^.

3 $${V}_{pot}^{2}=\frac{{C}_{k}{T}_{s}}{{C}_{d}{T}_{0}}(CAP{E}^{\ast }-CAP{E}^{b}),$$

where C_k_ and C_d_ are the exchange coefficient for enthalpy and the drag coefficient, respectively. *T*_*s*_ is SST and *T*_0_ is the mean outflow temperature at the level of neutral buoyancy of an air parcel lifted from saturation at the SST. The lower the outflow temperature is, the greater thermodynamic efficiency is expected. CAPE^*^ and *CAPE*^b^ are the convective available potential energy (CAPE) of the air displaced upward from saturation at sea level with reference to ambient air and the CAPE of the air at boundary layer, respectively.

## Electronic supplementary material

Supplementary Information

## Data Availability

The datasets for analysis are available, respectively, at https://climatedataguide.ucar.edu/climate-data/merged-hadley-noaaoi-sea-surface-temperature-sea-ice-concentration-hurrell-et-al-2008 for the observed SST, 10.5067/2E096JV59PK7 for the MERRA–2 atmospheric variables, and https://cds.nccs.nasa.gov/odas for OHC.
