# Supplementary material for: The Roles of Climate Change and Climate Variability in the 2017 Atlantic Hurricane Season
Source: Sci Rep. 2018 Nov 1;8:16172. doi: 10.1038/s41598-018-34343-5 (PMC6212430; doi:10.1038/s41598-018-34343-5)
Supplement: Supplementary file 1 — Supplementary Information [file 41598_2018_34343_MOESM1_ESM.docx]

**Supplementary Figures/Information**

**The Roles of Climate Change and Climate Variability in the 2017 Atlantic Hurricane Season**

**Young-Kwon Lim^1,2^, Siegfried D. Schubert^1,3^, Robin Kovach^1,3^,**

**Andrea M. Molod^1^, and Steven Pawson^1^**

^1^Global Modeling and Assimilation Office, NASA/GSFC, Greenbelt, Maryland

^2^Goddard Earth Sciences Technology and Research / I. M. Systems Group

^3^Science Systems and Applications, Inc., Lanham, MD

Second revision submitted to Scientific Reports

September 26, 2018

**Supplementary Figure 1**

**
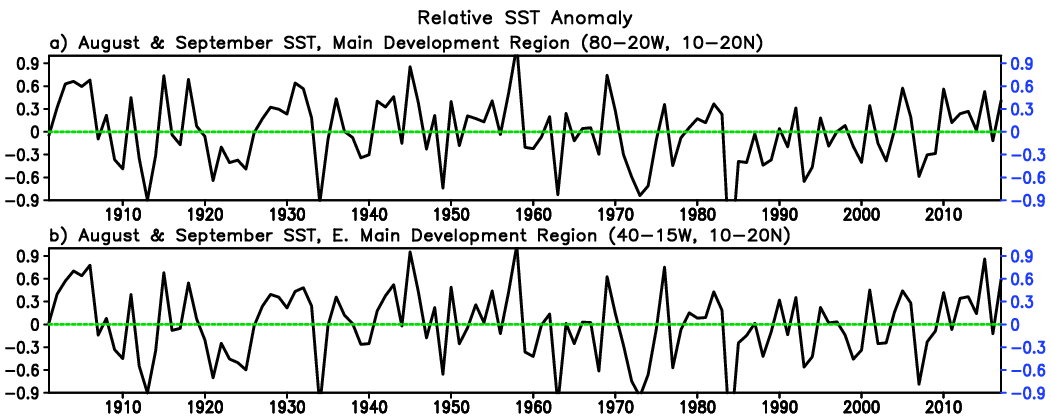
**

**Figure 1**. Time series of the August/September SST anomalies relative to the mean SST averaged over 0°–360°E, 30°S–30°N. The results are presented for the period 1901–2017 for the MDR (upper-panel) and EMDR (lower-panel)..

**Supplementary Figure 2**

The main features of the three leading modes (ENSO, AMM, and NAO) are briefly summarized here:

1. The El Niño mode (Fig. 2a) shows positive SST anomalies over the tropical eastern Pacific, with near zero or negative anomalies across the MDR^1^, indicating unfavorable conditions for the TC genesis over the North Atlantic. The PC time series and the Niño 3.4 SST index in Fig. 2d show the positive peaks during El Niño events (e.g., 1982, 1997, and 2015). The weak La Niña (or near neutral) conditions of August/September 2017 are manifested in the small amplitude of this PC.
2. The positive phase of the AMM mode (Fig. 2b) is characterized by positive SST anomalies over most of the Northern Atlantic covering the MDR^2,3^. Both 2005 and 2010 experienced large positive phases of the AMM (Fig. 2e) - years with the strongest Atlantic TC activity so far this century. 2017 is also characterized by a large positive phase of the AMM that contributed to a favorable environment for TC activity, but the magnitude is a little smaller than those for 2005 and 2010 (Fig. 2e).
3. The positive phase of the NAO-like mode (Fig. 2c) includes the well-known North-South tripole structure over the extra-tropical Atlantic^4^. A negative or near zero SST anomaly dominates the MDR, which is not favorable for strong TC activity. The negative phase of the NAO is known to be more favorable for TC genesis over this region^5^. While the weak TC activity coincide with the positive phase of this mode in 2013, the modest amplitude negative NAO in 2017 (PC in Fig. 2f) indicates that the NAO is likely to have had a positive impact on the TC activity.


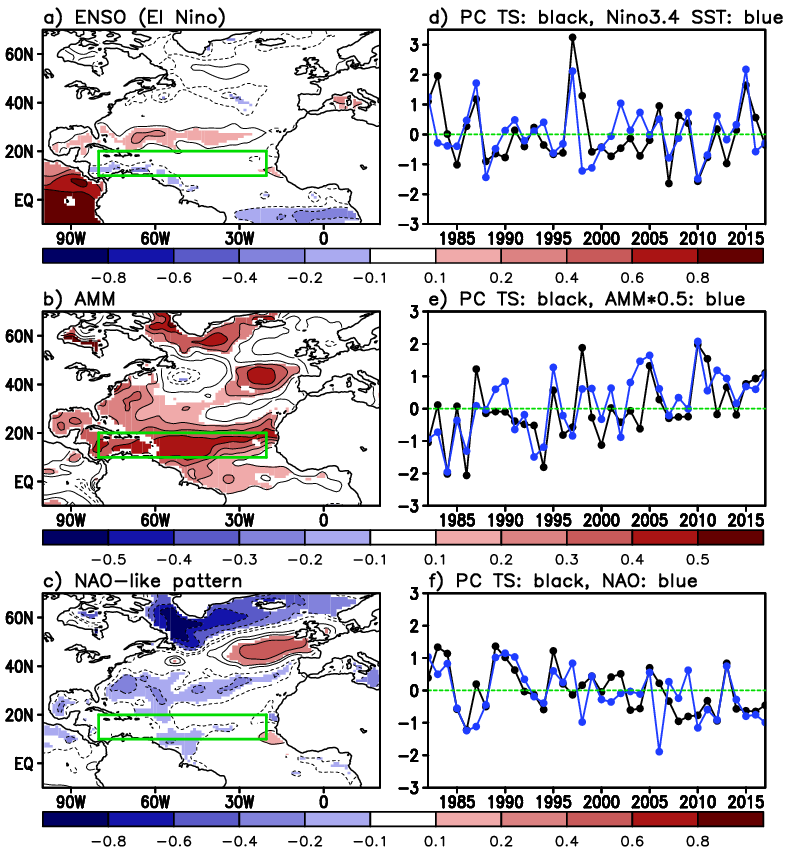


**Figure 2**. The first three REOFs of the detrended observed SST (°C) for August/September 1982–2017. The climate change signal included in the long-term upward trend has been first removed for the period 1901–2017 to solely investigate the climate variability, and the resulting SST for the period 1982–2017 is applied to the REOF analysis. From top to bottom, each panel represents the ENSO, the AMM, and the NAO-like mode. The left panels show distributions of non-normalized eigenvectors while the corresponding PC time series (black) are on the right. Eigenvector values statistically significant at 10 percent are shaded. Green boxes denote the Main Development Region (MDR). The sign convention corresponds to what is generally accepted to be the positive phases of these modes. Blue lines denoting official indices archived at NOAA/CPC are superimposed to indicate strong agreement with the PCs. Note that the frequent positive phase of the AMM in recent years (panel e) is associated with the Atlantic Multidecadal Oscillation that has been in the positive phase on decadal time scale since 1995.

**Supplementary Figure 3**

**
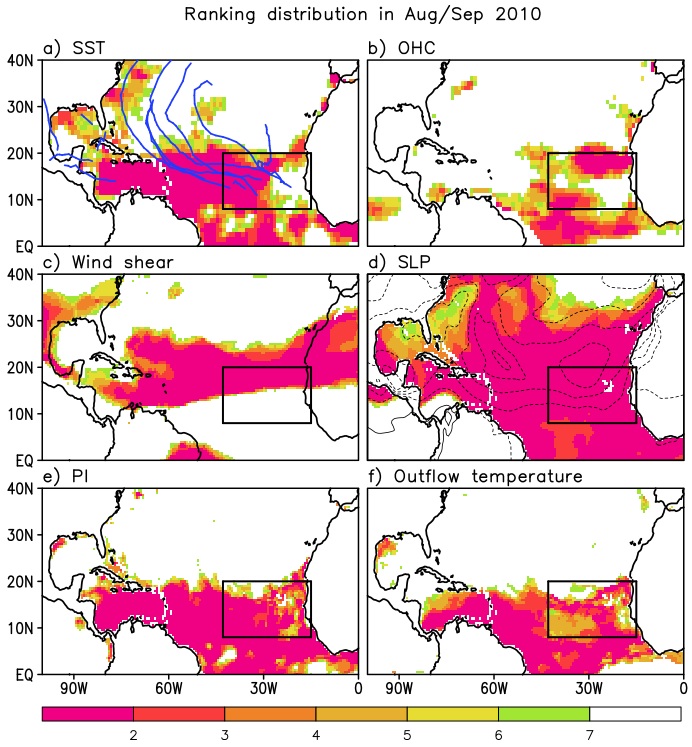
**

**Figure 3**. Same as Figure 4 in the main article but for the other extremely strong hurricane year that occurred in 2010. The ranking is calculated at each grid point for the years 1995-2017 - the recent period of above-average TC activity.

**Supplementary Figure 4**

**
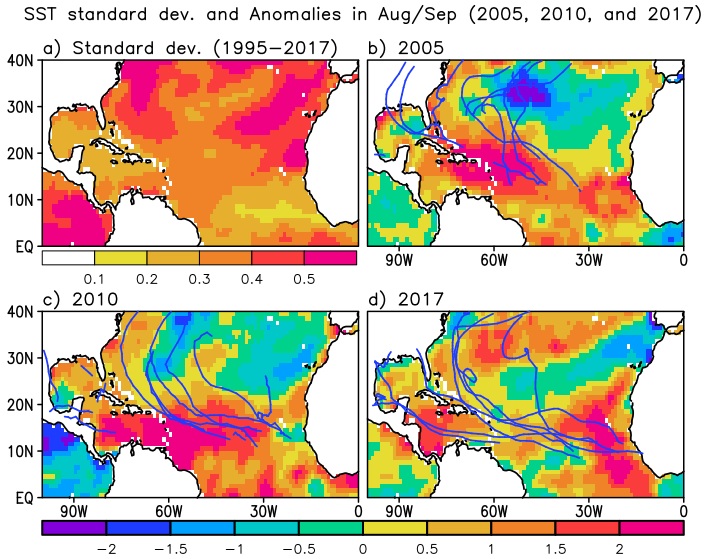
**

**Figure 4**. a) Distribution of standard deviation of the August/September mean SST over 1995–2017. Three other panels represent standardized anomaly distributions for 2005 (b), 2010 (c), and 2017 (d). Standardized anomaly is defined as the anomaly for a particular year divided by standard deviation. The blue lines in (b), (c), and (d) are the TC tracks observed in August/September each year.

**Supplementary Figure 5**

**
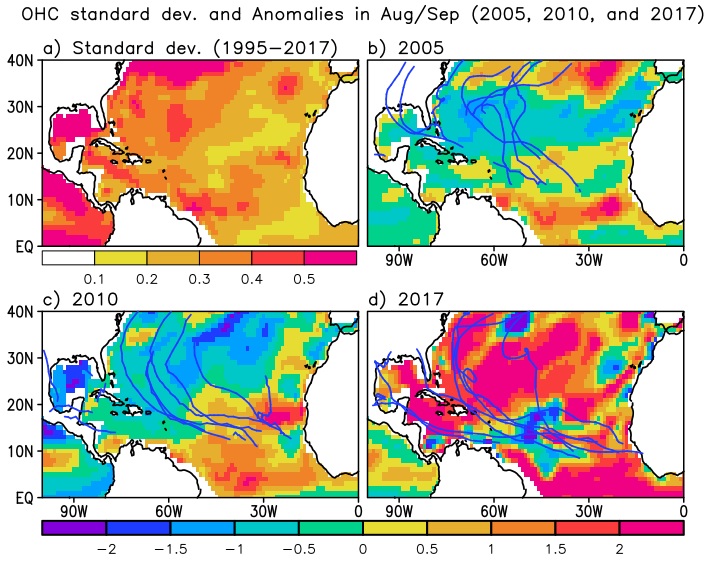
**

**Figure 5**. Same as Fig. 4 but for ocean heat content.

**Supplementary Figure 6**

**
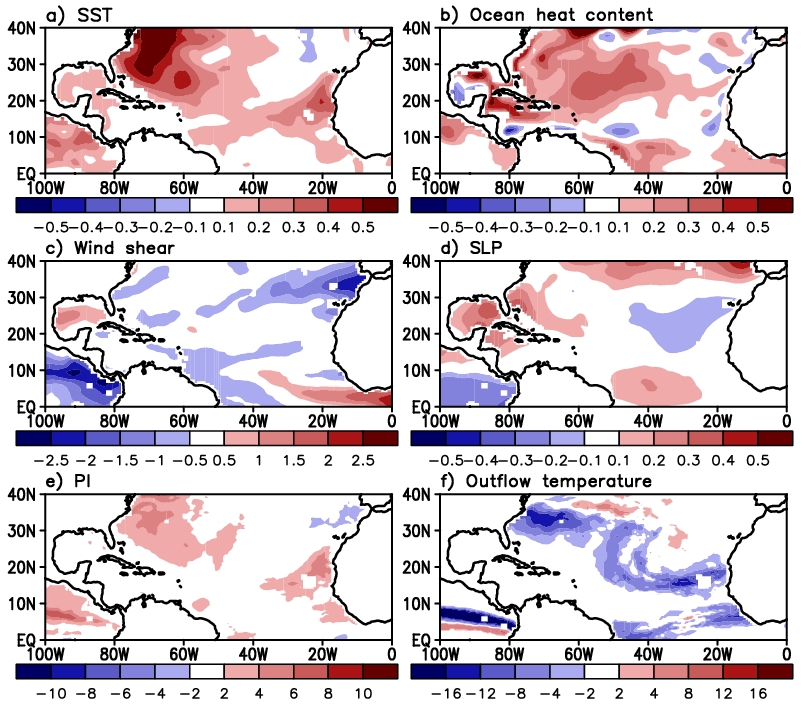
**

**Figure 6**. Distribution of the trend (per decade) over the recent above-average TC activity period 1995–2017. We note that, while the OHC trend computed from the GMAO ODAS (panel b) is generally similar to the OHC trend computed from the NOAA National Oceanic Data Center (NODC) data, there are differences that suggest there may be some uncertainty in the OHC estimates. One notable difference is that the trend values computed for this period in the GMAO ODAS OHC data (panel b) to a large extent reflect a recent substantial OHC increase that occurred near the end of global warming hiatus (around 2013), while the NOAA NODC OHC data show a more gradual (linear) increase over this time period.

**Supplementary Figure 7**

We calculate the AMM-associated patterns in key variables during AS 2017. Supplementary Figure 7 compares the anomaly fields (from the mean over 1995-2017, the recent period of above-average TC activity) with those determined by regressing the anomaly patterns against the AMM. The results show that the spatial distributions of the regressed anomalies tend to match the actual anomalies well, indicating an important role of the AMM. The spatial correlations over the North Atlantic basin (100°–15°W, 5°–50°N) for OHC and SLP are found to be 0.65 and 0.70, respectively. Figure 7b suggests that the enhancement of the SLP anomaly over the central mid-latitude Atlantic is associated with changes in the Azores high. The positive SLP anomaly there likely acts to produce TC tracks that extend westward into the Caribbean Sea, Gulf of Mexico, and the southeastern US, and then recurve along the western edge of the Atlantic high SLP system.

The AMM also contributes to the tropospheric shear and humidity anomaly, though the match between the regressed and actual anomaly is not as strong: the spatial correlations are 0.48 (for wind shear), and 0.45 (for humidity). Decreases in shear and increases in relative humidity over the MDR associated with the positive phase of the AMM is consistent with the results of ^3,6^. Additional confirmation that the key variables are connected more strongly with the AMM than with the ENSO or the NAO, is presented in Supplementary Figure 8 (see the time series and related discussion). The AMM is also more closely related to the interannual variation of the number of major hurricanes than either ENSO or the NAO (Fig. 8e).

**
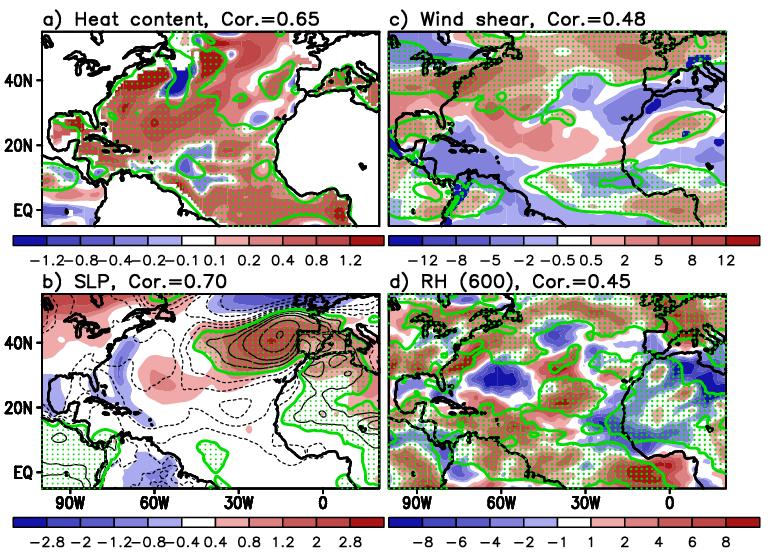
**

**Figure 7**. Shadings represent the observed anomaly of ocean heat content (10^22^J), SLP (mb), vertical wind shear (m s^-1^), and relative humidity (%) during August/September 2017 from the mean over 1995–2017. Green dots represent the area of positive regressed anomaly associated with the AMM. For wind shear, negative anomaly favors hurricanes. The regressed anomaly values are additionally contoured for the SLP (panel b). The thick green contours denotes the zero line of the regressed anomaly values. Spatial correlations between the actual anomaly and regressed anomaly over the Atlantic basin (100°–15°W, 5°–50°N) are shown above the each panel.

**Supplementary Figure 8**

We first examine the Genesis potential index (GPI)^7^, a widely used index that includes a number of quantities (e.g., SST, atmospheric shear, humidity, SLP, and lower level vorticity) to explain TC genesis activity. The top panel shows the year–to–year variations of the anomalous GPI over the MDR. The variations are remarkably coincident with the interannual variation of the AMM, denoted by the black line. The vertical wind shear (b), relative humidity (c), and tropical ocean heat content (d) are also found to co-vary with the AMM. The temporal correlations with the AMM are 0.52 (GPI), 0.68 (vertical wind shear × (–1)), 0.39 (relative humidity), and 0.70 (tropical ocean heat content), all of which are statistically significant at the 1% level, except for relative humidity. These correlations are considerably higher than those with El Niño and the NAO (compare correlations inside each panel). Because the AMM (and also the AMO) is related to an anomalous meridional SST gradient in the tropics and associated cross-equatorial circulation^3^, it is not surprising that the interannual variation of ocean heat content matches well the variation of the AMM with a relatively high correlation of 0.70 (Fig. 8d). The bottom panel (Fig. 8e) reveals that the AMM variation closely follows the number of major hurricanes in August–October each year (correlation=0.42), comparable to ENSO and better than the NAO.

**
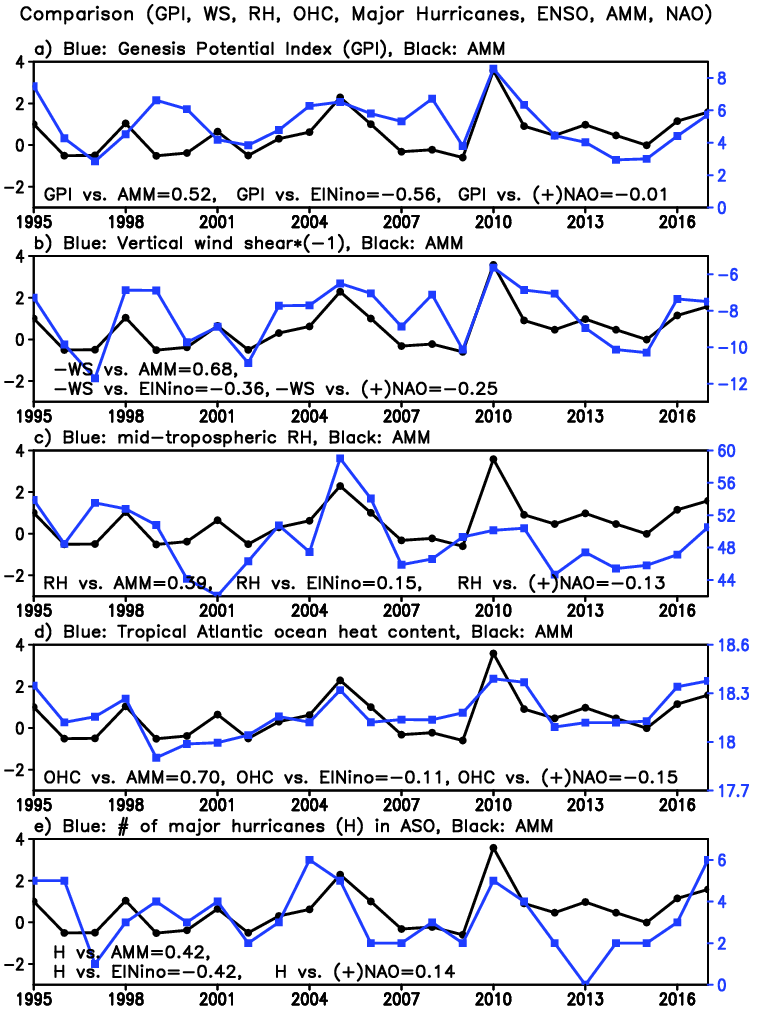
**

**Figure 8**. The first four panels: Interannual variation of the Genesis Potential Index (GPI), vertical wind shear multiplied by –1 (m s^-1^), mid-tropospheric relative humidity (600mb) (%), and ocean heat content (10^22^ J) over the Main Development Region in August–September over 1995–2017, all of which are denoted by blue lines. Time series in black represents the AMM time series over the same period. The bottom panel: Blue line represents the interannual variation of the number of major hurricanes during August–October each year whereas the black line is the AMM time series. Correlations between the five quantities (GPI, shear, humidity, ocean heat content, and major hurricane count) versus each climate mode are provided inside each panel.

**References**

1. Deser, C., Alexander, M. A., S.-P. Xie, S.-P. & Phillips, A. S. Sea surface temperature variability: Patterns and mechanisms. *Annu. Rev. Mar. Sci*. **2**, 115-143 (2010).
2. Chiang, J. C. H. & Vimont, D. J. Analagous meridional modes of atmosphere-ocean variability in the tropical Pacific and tropical Atlantic. *J. Climate* **17**, 4143-4158 (2004).
3. Vimont, D. J. & Kossin, J. P. The Atlantic meridional mode and hurricane activity. *Geophys. Res. Lett*. **34**, L07709 (2007).
4. Cassou, C., Deser, C., Terray, L., Hurrell, J. W. & Drévillon, M. Summer sea surface temperature conditions in the North Atlantic and their impact upon the atmospheric circulation in early winter. *J. Climate* **17**, 3349-3363 (2004).
5. Elsner, J. B. & Jagger, T. Prediction models for annual US Hurriane counts. *J. Climate* **19**, 2935-2952 (2006).
6. Lim, Y.-K., Schubert, S. D., Reale, O., Molod, A. M., Suarez, M. J. & Auer, B. M. Large-scale controls on Atlantic tropical cyclone activity on seasonal time scales. *J. Climate* **29**, 6727-6749 (2016).
7. Emanuel, K. A. & Nolan, D. S. Tropical cyclone activity and global climate. Proceedings, *The 26th Conference on Hurricanes and Tropical Meteorology.* Miami, FL, Amer. Meteor. Sci., 240-241 (2004).
